# Are non-lactose-fermenting *Escherichia coli* important diarrhoeal pathogens in children and adults?

**DOI:** 10.1099/acmi.0.000459.v3

**Published:** 2023-07-12

**Authors:** Bhawna Sharma, Vinay Modgil, Jaspreet Mahindroo, Ajay Kumar, Varpreet Kaur, Chandradeo Narayan, Ritu Verma, Balvinder Mohan, Neelam Taneja

**Affiliations:** ^1^​ Department of Microbiology, All India Institute of Medical Sciences, Bathinda, India; ^2^​ Department of Medical Microbiology, Postgraduate Institute of Medical Education and Research, Chandigarh, India

**Keywords:** adults, diarrhoea, *Escherichia coli*, enteroinvasive *Escherichia coli*, non-lactose-fermenting

## Abstract

**Introduction.:**

Diarrhoeagenic *Escherichia coli* (DEC) remains one of the major causes of acute diarrhoea episodes in developing countries. The percentage of acute diarrhoea cases caused by DEC is 30–40 % in these countries. Approximately 10% of *E. coli* isolates obtained from stool specimens have been reported to be non-lactose-fermenting (NLF). The available literature is sparse regarding the pathogenicity of NLF *E. coli* causing infectious diarrhoea.

**Aim.:**

We aimed to elucidate the importance of NLF *E. coli* in causing diarrhoea in both adults and children by detecting various DEC pathotypes among NLF *E. coli* in stool samples taken from gastroenteritis cases.

**Material and Methods.:**

A total of 376 NLF *E. coli* isolates from 3110 stool samples from diarrhoea/gastroenteritis patients were included in the study. Up to three NLF colonies that were not confirmed as *Vibrio cholerae *, *Aeromonas* spp., *Salmonella* spp. or *Shigella* spp., but were identified as *E. coli* using matrix-assisted laser desorption/ionization time-of-flight (MALDI-TOF), were carefully picked up from each MacConkey agar plate and then meticulously streaked onto freshly prepared, sterilized nutrient agar plates, and biochemical reactions were conducted. Multiplex PCR was conducted for the EAEC, EPEC, ETEC and EHEC pathotypes and PCR for the *ipaH* gene was conducted for EIEC. The disc diffusion method was used for antibiotic sensitivity testing.

**Results.:**

Using multiplex PCR and *ipaH* PCR, a total of 63 pathotypes of DEC were obtained, with EAEC being the most predominant (*n*=31) followed by EIEC (*n*=22), EPEC (*n*=8) and ETEC (*n*=2). To further differentiate EIEC from *Shigella *, additional biochemical tests were performed, including acetate utilization, mucate and salicin fermentation, and aesculin hydrolysis. Antimicrobial susceptibility testing (AST) showed that maximum resistance was seen against ciprofloxacin (82.5 %) followed by ampicillin (77.8 %) and cotrimoxazole (68.2 %), and minimum resistance was seen against ertapenem (4.8 %).

**Conclusion.:**

In our study two pathotypes (EAEC, EIEC) were predominant among NLF *E. coli* and these were not only important aetiological agents in children, but also in adults. Our study also sheds light on the epidemiology of EIEC, which is one of the most neglected DEC pathotypes, as hardly any microbiological laboratories process NLF *E. coli* for EIEC.

## Data Summary

All supporting data is provided in the manuscript.

## Introduction

Infectious diarrhoea plays an important role in morbidity and mortality worldwide, especially in children under 5 years old in the developing world [1–3]. Among the bacterial causes, diarrhoeagenic *Escherichia coli* (DEC) pathotypes, which cause a range of illnesses varying in severity from acute watery diarrhoea to dysentery, are the pivotal aetiological agents [4]. Based on their virulence and phenotypic characteristics, DEC are categorized into six main pathotypes, i.e. enteropathogenic *E. coli* (EPEC), enterotoxigenic *E. coli* (ETEC), enteroinvasive *E. coli* (EIEC), enteroaggregative *E. coli* (EAEC), Shiga toxin-producing *E. coli* (STEC) and diffusely adherent *E. coli* (DAEC).

Up to 10 % of isolates of *E. coli* have historically been stated to be slow or non-lactose fermenting (NLF), although the clinical implications of isolating these are unknown [5]. In most routine microbiology laboratories, only lactose-fermenting colonies of *E. coli* are further evaluated for DEC. NLF colonies, after *Shigella* and *Salmonella* are excluded, are not processed further for DEC pathotypes. Although a few studies have tried to elucidate the pathogenicity of NLF *E. coli,* the available literature are sparse regarding the pathogenicity of NLF *E. coli* causing infectious diarrhoea.

An important organism to look for will be enteroinvasive *E. coli* (EIEC), which tends to be invasive in colonic epithelial cells, where it causes an illness that is very similar to shigellosis. Biochemically, EIEC is also similar to *Shigella *, and very few biochemical tests can distinguish EIEC from *Shigella *. Therefore, in many cases the EIEC pathotype is misreported as *Shigella* [6]. EIEC also shares invasion plasmids with *Shigella *, hence molecular methods based on *ipaH* antigens are used for its detection. There are hardly any data available on the epidemiology of EIEC in the literature. We aimed to elucidate the importance of NLF *E. coli* in causing diarrhoea in both adults and children by detecting various DEC pathotypes (EIEC, EPEC, ETEC, EAEC, EHEC) among NLF *E. coli* obtained in stool samples from patients with gastroenteritis, and found that two DEC pathotypes, i.e. EIEC and EAEC, will be missed in a considerable proportion of stool specimens if NLF *E. coli* are not evaluated for pathogenicity.

## Methods

### Sample selection

**Fig. 1.**
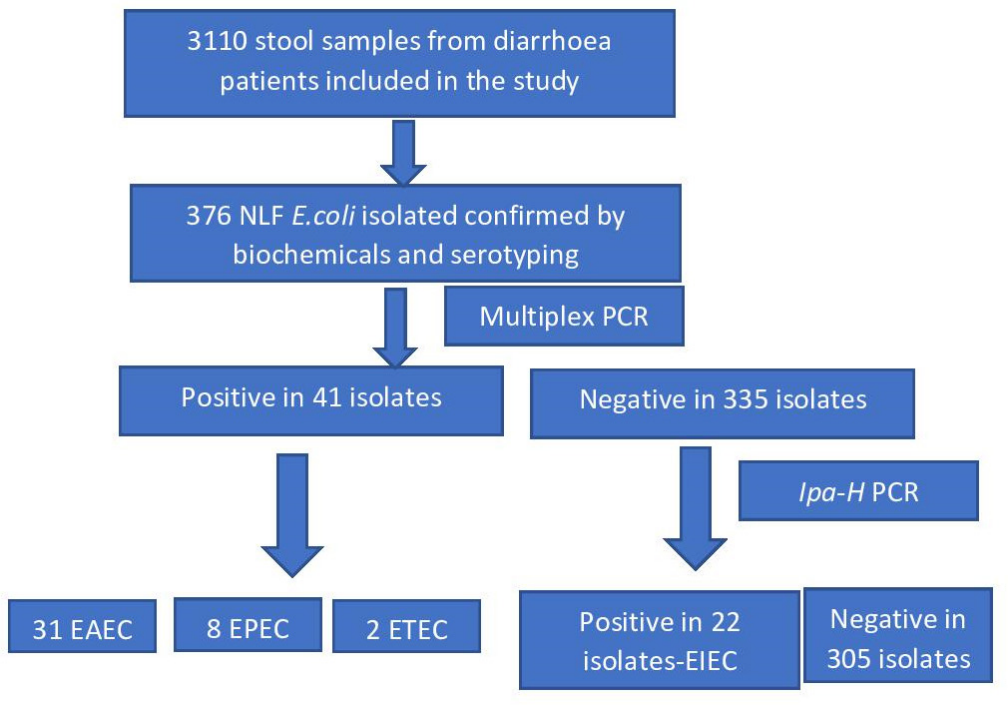
Flow chart showing the scheme of the study.

A total of 376 NLF *E. coli* isolates were then isolated from 3110 stool samples from gastroenteritis or diarrhoea patients from the out-patient department (OPD) and the in-patient department (IPD), covering all age groups from different wards of PGIMER, Chandigarh from January 2019 to April 2021 (Fig. 1). Clinical data for the diarrhoeal episodes and other related symptoms were collected for each case. It was mandatory for each patient to provide written, informed consent.

### Sample collection and processing

Stool samples from the gastroenteritis or diarrhoea patients were collected in sterile containers, inoculated into a transport medium, Cary Blair medium, and sent to the laboratory via cold chain. They were cultured for the presence of *Aeromonas* sp., *Vibrio cholerae *, *Salmonella* spp., *Shigella* spp. and *E. coli* using standard procedures [7]. Samples were then inoculated onto selenite F broth, MacConkey agar, xylose lysine deoxycholate (XLD) agar, ampicillin blood agar, thiosulfate–citrate–bile salts–sucrose agar (TCBS) agar and alkaline peptone water (APW) and then incubated at 37 °C for 18–24 h. The organisms found were then identified using standard biochemicals [7] and matrix-assisted laser desorption/ionization time-of-flight (MALDI-TOF), which was performed on a MALDI Microflex LT mass spectrometer (Bruker Daltonik GmbH, Bremen, Germany). Up to three NLF colonies that were not confirmed as *Aeromonas* spp., *V. cholerae *, *Salmonella* spp., or *Shigella* spp., but were identified as *E. coli* on MALDI-TOF, were selected from each MacConkey agar plate and streaked onto fresh, sterilized nutrient agar and stored in trypticase soy broth containing 15% glycerol at −80 °C.

### Biochemical reactions

**Fig. 2.**
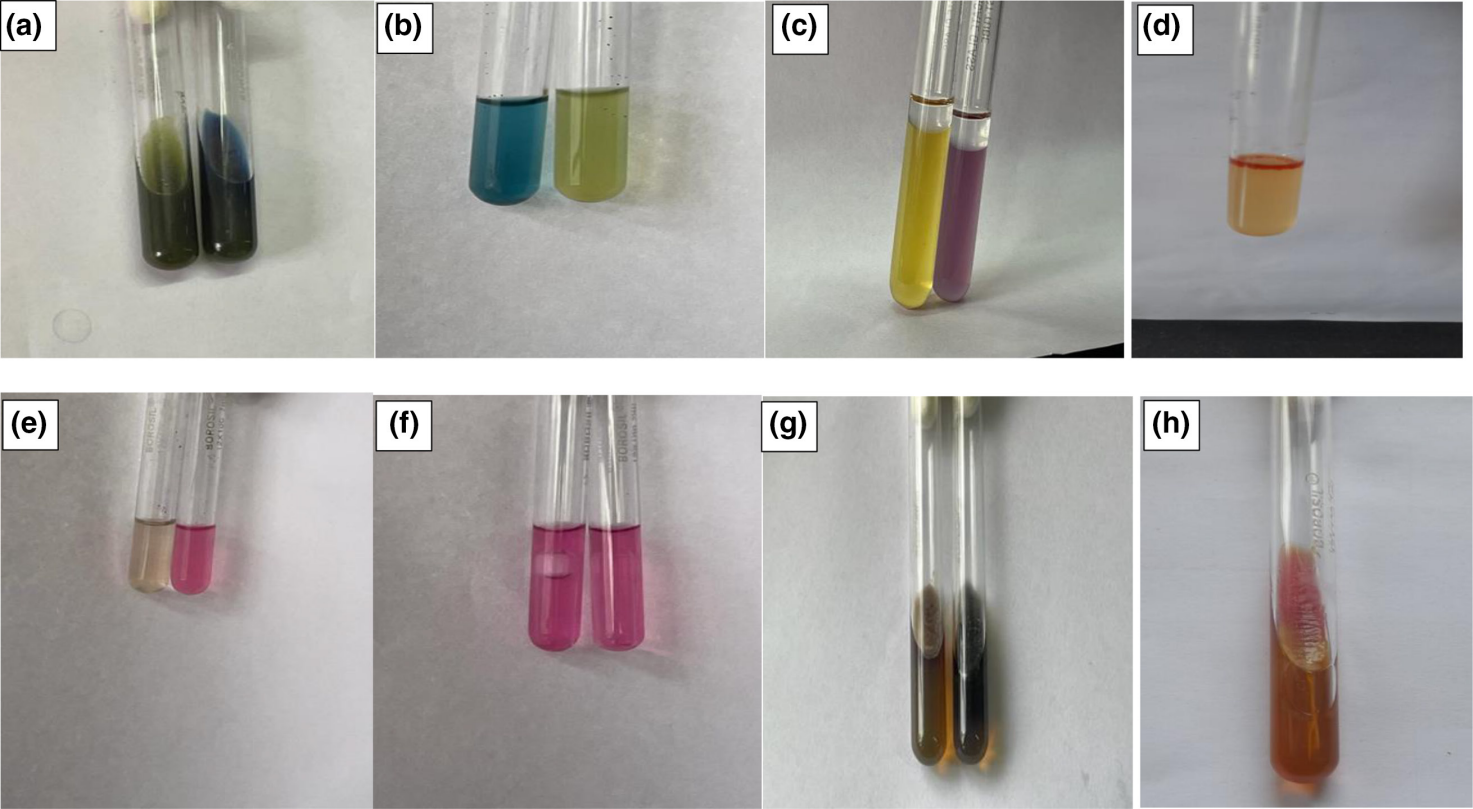
Biochemical reactions for EIEC. (a) Acetate utilization test (green, negative; blue, positive). (b) Mucate fermentation (blue, positive; yellow, negative). (c) Lysine derboxylase (yellow, control; purple, positive). (d) Positive indole test. (e) Salicin fermentation (colourless, negative; pink, positive). (f) Glucose fermentation with gas/without gas. (g) Bile aesculin hydrolysis (yellow, negative; black, positive). (h) Triple sugar iron (K/A with gas formation).

Biochemical reactions were conducted for all the putative NLF *E. coli* that were negative for serotyping (Denka Seiken Co. Ltd, Japan) of *Shigella* to differentiate them from *Shigella* species [8]. These included triple sugar iron, motility, indole test, lysine decarboxylase, citrate utilization, glucose and xylose fermentation with Durham’s tube, mannitol fermentation and o-nitrophenyl-β-d-galactopyranoside (ONPG) test (Fig. 2).

### Extraction of DNA from confirmed *E. coli*

DNA from each confirmed NLF *E. coli* isolate was extracted via the heat extraction method as described by Dilhari *et al*. [9]

### Identification of DEC genes

A multiplex PCR for enterotoxins [heat-labile (LT) and heat-stable (ST)], EPEC (Eae) protein bundle-forming protein (Bfp), Shiga toxins (Stx1, Stx2), VTcom for EHEC and (CVD432) for EAEC was performed using published primers [9–14] and the protocol shown in Table 1.

**Table 1.** List of primers, their sequences and the size of the amplified products used in this study

Target gene	Primer sequence (5’–3’)	Primer designation	PCR product size (bp)	DEC pathotype	Reference
*Bfp*	F: GGAAGTCAAATTCATGGGGGTAT R: GGAATCAGACGCAGACTGGTAGT	Bfp	300	EPEC	[10]
*Eae*	F: TCAATGCAGTTCCGTTATCAGTT R: GTAAAGTCCGTTACCCCAACCTG	Eae	482	EPEC	[10]
*Elt*	F: ACGGCGTTACTATCCTCTC R: TGGTCTCGGTCAGATATGTG	LT	273	ETEC	[11]
*CVD432*	F: CTGGCGAAAGACTGTATCAT R: AATGTATAGAAATCCGCTGTT	pCVD432	630	EAEC	[12]
*estA1*	F: TCTTTCCCCTCTTTTAGTCAG R: ACAGGCAGGATTACAACAAAG	STp	166	ETEC	[11]
*estA2-4*	F: TTCACCTTTCCCTCAGGATG R: CTATTCATGCTTTCAGGACCA	STh	120	ETEC	[11]
*stx1*	F: CAGTTAATGTGGTGGCGAAGG R: CACCAGACAATGTAACCGCTG	Stx1	348	EHEC	[10]
*stx2*	F: ATCCTATTCCCGGGAGTTTACG R: GCGTCATCGTATACACAGGAGC	Stx2	584	EHEC	[10]
*stx1 +stx2*	F: GAGCGAAATAATTTATATGTG R: TGATGATGGCAATTCAGTAT	VTcom	518	ETEC	[13]
*ipaH*	F: GAAAACCCTCCTGGTCCATCAGG R:GCCGGTCAGCCACCCTCTGAGAGTAC	*ipaH*	437	EIEC	[14]

F, forward primer; R, reverse primer.

### PCR conditions for DEC genes

In multiplex PCR reactions two or more pairs of primers were used. All the PCR reactions were performed in 20 µl final volume containing 0.5 µl of the template DNA, 1 µl of DNA polymerase, 0.2 mM of each dNTP, 1.5 mM of MgCl_2_ and 10 µM of each primer. The thermocycling conditions for all the PCRs were as follows: 95 °C for 2 min, 95 °C for 15 s, 52 °C for 8 s and 10 s at 72 °C for 30 cycles, with a final 2 min extension at 72 °C. PCR products were subsequently analysed on a 1.5 % agarose gel electrophoresis in Tris–borate–EDTA with ethidium bromide (EtBr) staining.

### Defining criteria for EIEC

NLF *E. coli* isolates negative by the above DEC multiplex PCR were subsequently tested for identification of EIEC using the *ipaH* gene (Table 1).

### PCR for *ipaH* gene

In a single PCR reaction, one pair of primers was used. PCR reaction was conducted at a final volume of 25 µl comprising 0.5 µl DNA, 1 µl DNA polymerase, 2.6 mM of each dNTP, 1.5 mM of MgCl_2_. Primer was used at 10 µM concentration [15].

### The thermocycling parameters for PCR (*ipaH* gene)

The thermocycling parameters for the *ipaH* gene in PCR were as follows in the thermal cycling programme: initial denaturation at 95 °C for 5 min, 1 cycle; denaturation at 94 °C for 30 s, 35 cycles; annealing at 58 °C for 30 s, 35 cycles; extension at 72 °C for 1 min, 35 cycles; final extension at 72 °C for 7 min, 1 cycle; and holding at 4 °C, 1 cycle. The amplification products were electrophoresed through a 2 % agarose gel and visualized with UV transilluminator after EtBr staining. A 100 bp DNA ladder was used as a molecular size marker in the gel.

### Antibiotic sensitivity testing

Antibiotic sensitivity testing was performed with the disc diffusion method for the following antibiotics: cefepime (15 mg), piperacillin–tazobactam (10 mg), ertapenem (10 mg), ampicillin (10 mg), ciprofloxacin (5 mg), gentamicin (10 mg), cotrimoxazole (25 mg), cefoxitin (30 mg), amikacin (30 mg), imipenem (10 mg), levofloxacin (5 mg) and ceftriaxone (30 mg) as per the Clinical and Laboratory Standards Institute (CLSI) guidelines [16]. Multidrug resistance was described as acquired resistance to three or more antimicrobials from different antimicrobial classes tested.

### Serotyping for *E. coli* O serotypes

All EAEC and EIEC isolates were sent to the National *Salmonella* and *Escherichia* Centre, Central Research Institute, Kasauli (HP), India for *E. coli* O serotyping [17].

## Results

**Fig. 3.**
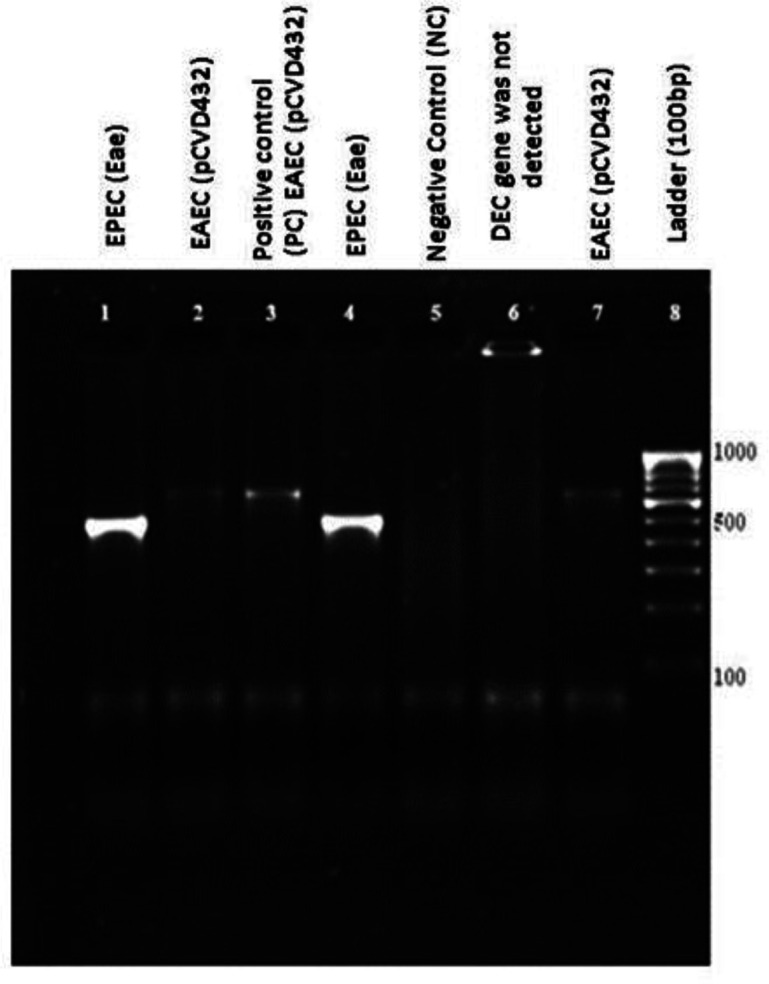
A representative gel electrophoresis profile of DEC genes. Lane 1, EPEC (*eae*); lane 2, EAEC (*pCVD432*); lane 3, positive control (PC); lane 4, EPEC (*eae*); lane 5, negative contol (NC); lane 6, DEC gene was not detected; lane 7, EAEC (*pCVD432*), lane 8, ladder (100 bp).

**Fig. 4.**
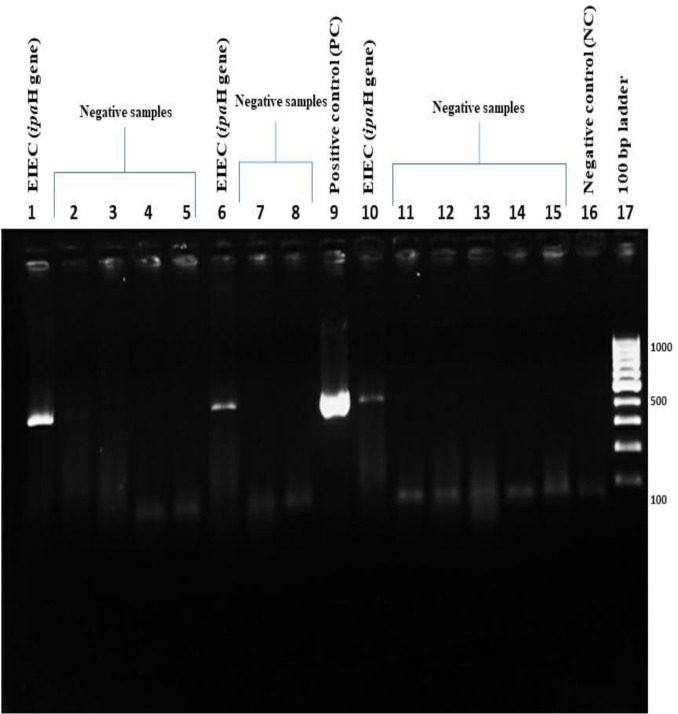
A representative gel electrophoresis profile of the *ipa*H gene. Lane 1, EIEC (*ipa*H); lanes 2, 3, 4, 5, *ipa*H gene was not detected; lane 6, EIEC ; lane 7, 8, *ipa*H gene was not detected; lane 9, positive control (PC); lane 10, EIEC (*ipa*H); lanes 11, 12, 13, 14, 15, *ipa*H gene was not detected; lane 16, negative control (NC); lane 17, ladder (100 bp).

Out of a total of 3110 stool samples, 376 isolates of NLF *E. coli* were obtained from 376 patients suffering from gastroenteritis with an average age of 31±2 years. Out of 376 patients, 199 were male and 177 were female, while 180 patients were from the OPD and 196 were from the IPD. Biochemical reactions, along with serotyping for *Shigella *, were conducted for all 376 isolates to differentiate them from *Shigella* species (Table 2, Fig. 2). All of the isolates were negative for indole production, on triple sugar iron agar all the isolates showed K/A reaction with gas production, lysine production was positive for all the isolates and out of all 4.8 % (18) were non-motile. Using multiplex PCR (Fig. 3) and *ipaH* PCR (Fig. 4), a total of 63 pathotypes of DEC were obtained, with EAEC being the most predominant (*n*=31) followed by EIEC (*n*=22), EPEC (*n*=8) and ETEC (*n*=2). Details of demographic profiles and clinical presentation of cases from which DEC were isolated are given in Table 3. The overall average age from the samples yielding DEC in our study was 31±2 years, which was similar for EAEC (31.6 years) and ETEC (30.4 years). The average age for EIEC was 20.7 years and for EPEC it was 11 years. A total of 57 % (36/63) patients have been hospitalized.

**Table 2.** Biochemical reactions of NLF *E. coli* included in the study

Isolates	TSI	Indole		ONPG		Motility		Lysine decarboxylase		Citrate utilization		Glucose fermentation with gas	Mannitol fermentation		Xylose fermentation with gas
		+	−	+	−	Motile	Non-motile	+	−	+	_		+	−	
**NLF *E. coli *** (**376**)	**K/A with gas**	**376**	**0**	**374**	**2**	**358**	**18**	**376**	**0**	**0**	**376**	**376**	**374**	**2**	**376**

**Table 3.** Demographic profile and clinical profile of diarrhoeagenic *E. coli* (DEC)

Clinical characteristics	Average age (years)	Sex		Opd/IPD		Diarrhoea		Diarrhoea with dysentery *n* (%)	Fever *n* (%)	Abdominal pain *n* (%)
		Male *n* (%)	Female *n* (%)	OPD *n* (%)	IPD *n* (%)	Acute *n* (%)	Chronic/persi stent *n* (%)			
**EAEC (31**)	31.6	19 (61.2 %)	12 (38.7 %)	µ (32.2 %)	21 (67 %)	12 (38.7 %)	18 (58 %)	3 (9 %)	4 (12.9 %)	10 (32.2 %)
**EIEC (22**)	20.7	15 (68.2 %)	7 (31.8 %)	10 (45.5 %)	12 (54.5 %)	10 (45.5 %)	12 (54.5 %)	9 (40.1 %)	17 (77.3 %)	20 (90.9 %)
**EPEC (8**)	11	5 (62.5 %)	3 (37.5 %)	4 (50 %)	2 (25 %)	5 (62.5 %)	3 (37.5 %)	0	5 (62.5 %)	6 (75 %)
**ETEC (2**)	30.4	1 (50 %)	1 (50 %)	1 (50 %)	1 (50 %)	2 (100 %)	0	0	1 (50 %)	1 (50 %)

The biochemical profiles of these DEC pathotypes are shown in Table 4. To further differentiate EIEC from *Shigella *, additional biochemicals were performed, including fermentation of mucate, acetate utilization, and salicin fermentation and aesculin hydrolysis (Table 5). DEC were serotyped to determine the O antigens of EIEC and EAEC. Out of a total of 22 isolates of EIEC, *E. coli* O serotypes were detected in 15 isolates, and 7 strains were untypeable. The most common serotype was O120, present in four isolates, followed by O98 and O7, each in three isolates. Among 31 EAEC isolates, the most commonly encountered serotypes were O22, O88 and O149 (*n*=4), followed by O17 (*n*=3) and O11 (*n*=3); 2 isolates were of the O35 serotype, 2 were O84, and a single isolate each were O126, O128, O49 and O8 (Table 5). Antimicrobial susceptibility testing (AST) showed that maximum resistance was seen against ciprofloxacin (82.5 %) followed by ampicillin (77.8 %) and cotrimoxazole (68.2 %), while minimum resistance was seen against ertapenem (4.8 %). Among the different DEC pathotypes, significant numbers were only obtained for EAEC (31) and EIEC (22) to comment on the resistance pattern. Among EAEC, maximum resistance was seen against ampicillin (87.1 %) and ciprofloxacin (87.1 %), followed by ceftriaxone (74.2 %) and cotrimoxazole (64.5 %). Among EIEC strains, maximum resistance was seen against ciprofloxacin (81.8 %) and cotrimoxazole (81.8 %), followed by ampicillin (72.7 %). Statistical analysis was only performed between EAEC and EIEC by using Fischer’s exact test. Statistical analysis was performed using GraphPad Prism v.6.0 (GraphPad Software, La Jolla, CA, USA) and a *P*-value <0.05 was taken as significant. The antimicrobial resistance pattern of DEC is presented in Table 6.

**Table 4.** Biochemical reactions of DEC pathotypes

DEC (64)	TSI	Indole		ONPG		Motility		Ornithine decarboxylase		Lysine decar -boxylase		Arginine dihydrolase		Citrate utilization	Glucose fermentation with gas	Mannitol fermentation	
		+	−	+	−	Motile	Non- motile	+	−	+	−	+	−			+	−
**ETEC (2**)	**K/A with gas**	2	0	2	0	2	0	1	1	2	0	0	2	−	+	2	0
**EPEC (8**)	**K/A with gas**	8	0	8	0	8	0	3	20	8	0	3	5	−	+	8	0
**EAEC (31**)	**K/A with gas**	31	0	31	0	28	3	10	13	31	0	15	16	−	+	31	0
**EIEC (22**)	**K/A with gas**	22	0	21	2	4	18	10	12	22	0	6	16	−	+	21	1

**Table 5.** *
Escherichia coli* O serotyping and biochemicals for EIEC

Serotype	Mucate fermentation		Acetate utilization		Salicin fermentation		Esculin hydrolysis	
	Positive	Negative	Positive	Negative	Positive	Negative	Positive	Negative
**O120** (***n*=4**)	3	1	4	0	4	0	4	0
**O98** **(*n*=3)**	3	0	3	0	3	0	3	0
**O7** **(*n*=3)**	1	2	3	0	3	0	2	1
**O26** **(*n*=1)**	1	0	1	0	0	1	1	0
**O84** **(*n*=1)**	1	0	1	0	1	0	1	0
**O86** **(*n*=1)**	0	1	1	0	1	0	1	0
**O105** **(*n*=1)**	1	0	1	0	1	0	1	0
**O18** **(*n*=1)**	1	0	1	0	1	0	1	0
**Untypeable** **(*n*=7)**	5	2	7	0	6	1	4	3

**Table 6.** Antimicrobial resistance in DEC pathotypes Statistically significant (*P*<0.05) when antibiotic resistance among EAEC and EIEC was compared.

Antibiotic	Total, *n*=63 (%)	ETEC, *n*=2 (%)	EPEC, *n*=8 (%)	EAEC, *n*=31 (%)	EIEC, *n*=22 (%)	*P*-value
Ampicillin	49 (77.8 %)	1 (50 %)	5 (62.5 %)	27 (87.1 %)	16 (72.7 %)	0.29
Ciprofloxacin	52 (82.5 %)	1 (50 %)	6 (75 %)	27 (87.1 %)	18 (81.8 %)	0.70
Cefixime	16 (25.4 %)	0	3 (37.5 %)	3 (9.7 %)	10 (45.5 %)	0.004*
Cefoxitin	17 (27 %)	0	2 (25 %)	6 (19.4 %)	9 (40.9 %)	0.12
Ceftriaxone	41 (65.1 %)	1 (50 %)	4 (50 %)	23 (74.2 %)	13 (59 %)	0.37
Amikacin	9 (14.3 %)	0	2 (25 %)	4 (12.9 %)	3 (13.6 %)	1.0
Gentamicin	16 (25.4 %)	0	2 (25 %)	10 (32.2 %)	4 (18.2 %)	0.34
Imipenem	9 (14.3 %)	0	1 (12.5 %)	5 (16.1 %)	3 (13.6 %)	1.0
Ertapenem	3 (4.8 %)	0	1 (12.5 %)	1 (3.2 %)	1 (4.5 %)	1.0
Levofloxacin	27 (42.9 %)	1 (50 %)	2 (25 %)	12 (38.7 %)	12 (54.5 %)	0.28
Piperacillin–tazobactam	10 (15.9 %)	0	1 (12.5 %)	6 (19.3 %)	3 (13.6 %)	0.72
Cotrimoxazole	43 (68.2 %)	1(50 %)	4 (50 %)	20 (64.5 %)	18 (81.8 %)	0.22

## Discussion

Among children <5 years old in developing countries, diarrhoeagenic *Escherichia coli* (DEC) are responsible for ~30–40% of acute diarrhoea episodes [18], and are also an important cause of both sporadic cases and diarrhoeal outbreaks all over the world [19]. DEC contain and express various virulence factors that enable them to exhibit pathogenicity. NLF colonies are usually processed for *Salmonella *, *Shigella* and *Vibrio cholerae *. Although NLF *E. coli* are commonly isolated, they are usually not processed further. Moreover, *Shigella* are especially difficult to differentiate from NLF *E. coli* due to similar colony characters. MALDI-TOF also cannot differentiate between these two species. Therefore, reliance is placed on biochemicals such as indole, ONPG, lysine decarboxylase, acetate utilization and mucate fermentation and agglutination by antisera. *Shigella* strains are always lysine decarboxylation-negative, produce indole variably, are acetate utilization- and mucate fermentation-negative and ONPG-negative, except 15% cases of *Shigella dysentriae* type 1, all *Shigella sonnei* and 8% of *Shigella boydii* [8]. There is very little in the literature regarding the pathogenicity of NLF *E. coli *, as most of the studies focus on lactose-fermenting *E. coli *. Diarrhoeal disease data across the developing world focus on the paediatric population and very few studies from India have included adults [3, 20, 21]. Hence, to fill this gap the present study assessed the importance of NLF *E. coli* in stool specimens in diarrhoea cases across all age groups.

In the present study, 376 (12 %) NLF *E. coli* were obtained from 3110 stool samples, which yielded a total of 63 (16.7 %) strains of DEC. Similar to our study, Hossaine *et al.* [22] studied 74 (16 %) NLF *E. coli* in 460 diarrhoeal stools from children <5 years of age in Bangladesh and found 24 (32 %) strains of DEC. In our study, EAEC and EIEC were the predominant pathotypes in contrast to the study by Hossaine *et al.,* where EPEC followed by EAEC were the most common [22]. This difference could be because of the different age groups included in both of the studies. The average age of patients from whom DEC were isolated was 31.6 years for EAEC and 20.7 for EIEC. Our study highlights the role of EAEC and EIEC as a potential cause of diarrhoea not only in paediatric patients but also in adult patients. Moreover, both these pathotypes caused severe illness requiring hospitalization (67 % in EAEC and 52 % in EIEC).

Extensive geographical variations are observed in the prevalence of EAEC across the world and even in different regions of India [22–24]. Most of the previous studies on EAEC have only included paediatric patients [22, 25] and there is much less in the literature regarding the role of EAEC in the adult population [25–27]. In our previous study on lactose-fermenting *E. coli *, EAEC was present in a higher proportion (11.4 %), followed by EPEC (6.2 %) and ETEC (4.9 %), but these samples were not processed for EIEC and only paediatric patients (<10 years age) were included [28]. A recent study by Jensen *et al.* investigated the prevalence of EAEC in adult Danish patients suffering from diarrhoea and healthy controls, concluding that asymptomatic carriage of EAEC is not common in the Danish adult patients (1.2 %) as compared to children (10.5 %), whereas in symptomatic diarrhoeal patients, EAEC was detected in 4.6 % of patients and the median age was 34 years [26]. Similarly, in another study from Brazil, an increasing trend of isolation of EAEC in diarrhoeal stools in the adult population (57.9 %) as compared to children (42.1 %) was observed [27]. An interesting clinical presentation in our study was that 9.7 % of patients presented with dysentery and 56 % of EAEC cases presented with chronic/persistent diarrhoea. Jensen *et al.* in their study found that 39.7 % of EAEC patients had chronic diarrhoea and 15 % had bloody diarrhoea, similar to our study [26]. EAEC is known to cause intestinal inflammation, as evidenced by several studies in which the presence of faecal lactoferrin and proinflammatory cytokines, notably interleukin (IL)−8, was observed [29–35]. A growing number of studies have supported the association of EAEC with persistent/chronic diarrhoea in malnourished children and individuals with HIV infection in developing countries [36–38]. However, in our study, although 18 patients presented with persistent diarrhoea, none of them were immunocompromised and 10 of them were adult patients.

The next most common DEC pathotype in our study was EIEC, which shares an invasion plasmid antigen with *Shigella* [39, 40]. Based on the *ipaH* gene expression alone, it is difficult to differentiate between *Shigella* and EIEC [39]. The few biochemical properties that enable differentiation of *E. coli* and *Shigella* spp. are acetate utilization and mucate fermentation. EIEC may be positive for one or both of the properties, while *Shigella* strains are negative for both [8]. In our study all of the EIEC strains(*n*=22) were positive for either one (*n*=6) or both (*n*=16) of them. In addition, EIEC share the ability to produce gas from glucose and fermentation of xylose with other *E. coli* pathotypes . Salicin fermentation and aesculin hydrolysis may also be useful to differentiate between *Shigella* and EIEC [41]. *Shigella* is unable to ferment salicin and hydrolyze aesculin. In our study, 91 % of EIEC strains were positive for salicin fermentation and 82 % hydrolyzed aesculin.

The clinical illness caused by EIEC resembles *Shigella* and is characterized by abdominal cramps, diarrhoea, vomiting, fever, chills and a generalized malaise [39]. In our study only 39 % of EIEC patients presented with dysentery and 74 % of patients presented with other features of invasiveness, such as fever and abdominal cramps. A highlight of our study was the demonstration of EIEC causing chronic diarrhoea (56 %). We described for the first time such a presentation associated with EIEC infection. Usually, EIEC are only suspected in stool samples with mucus and blood, and not in other diarrhoeal cases, suggesting that the actual prevalence of EIEC may be higher than has been indicated in previous studies [39, 42].

Antibiotics may be required to treat invasive infections and persistent/chronic diarrhoea. Similar to other studies from India and Bangladesh, DEC pathotypes exhibited alarming rates of resistance against widely used antimicrobials – ciprofloxacin (82.5 %), ampicillin (77.8 %) and cotrimoxazole (68.2 %) – and minimum resistance was seen against ertapenem (4.8 %) [43–46]. Among the different DEC pathotypes, significant numbers were only obtained for EAEC (31) and EIEC (22) to make a comparison. The difference in the resistance pattern among EAEC and EIEC strains was not statistically significant, except for cefixime, as 45.5 % of EIEC isolates were resistant to cefixime as compared to 9.7 % EAEC isolates, (*P*-value=0.004). Very few studies to date have reported antibiotic resistance in EIEC strains [46, 47]. Chellapandi *et al.* have also reported high rates of resistance in their EIEC isolates among the northeast Indian population [47]. This resistance is concerning and needs to be monitored at the community level.

## Conclusion

This study is to the best of our knowledge the first one to highlight the importance of NLF *E. coli* in the aetiology of diarrhoea and gastroenteritis. In our study two pathotypes (EAEC, EIEC) were predominant among NLF *E. coli* and these are not only important aetiological agents in children, but also in adults. Our study also sheds light on the epidemiology of EIEC, which is the most neglected pathotype, as hardly any microbiological laboratories process NLF *E. coli* for EIEC. Moreover, in our study 56 % of patients were admitted, highlighting the importance of severity of illness caused by NLF *E. coli *.

## References

[1] Kotloff KL, Blackwelder WC, Nasrin D, Nataro JP, Farag TH (2012). The Global Enteric Multicenter Study (GEMS) of diarrheal disease in infants and young children in developing countries: epidemiologic and clinical methods of the case/control study. Clin Infect Dis.

[2] Mladenova Z, Steyer A, Steyer AF, Ganesh B, Petrov P (2015). Aetiology of acute paediatric gastroenteritis in Bulgaria during summer months: prevalence of viral infections. J Med Microbiol.

[3] Shrivastava AK, Kumar S, Mohakud NK, Suar M, Sahu PS (2017). Multiple etiologies of infectious diarrhea and concurrent infections in a pediatric outpatient-based screening study in Odisha, India. Gut Pathog.

[4] Saeed A, Abd H, Sandstrom G (2015). Microbial aetiology of acute diarrhoea in children under five years of age in Khartoum, Sudan. J Med Microbiol.

[5] Fernández-Castané A, Vine CE, Caminal G, López-Santín J (2012). Evidencing the role of lactose permease in IPTG uptake by *Escherichia coli* in fed-batch high cell density cultures. J Biotechnol.

[6] Lan R, Alles MC, Donohoe K, Martinez MB, Reeves PR (2004). Molecular evolutionary relationships of enteroinvasive *Escherichia coli* and *Shigella* spp. Infect Immun.

[7] Koneman E, Giovanniello O, Klajn D, Preciado M (2008). DiagnosticoMicrobiologico., Madrid España.

[8] Bopp CA, Brenner FW, Fields PI, Wells JG, Strockbine NA, Murray PR, Baron EJ, Jorgensen JH, Pfaller MA, Yolken RH (2003). Manual of Clinical Microbiology.

[9] Dilhari A, Sampath A, Gunasekara C, Fernando N, Weerasekara D (2017). Evaluation of the impact of six different DNA extraction methods for the representation of the microbial community associated with human chronic wound infections using a gel-based DNA profiling method. AMB Express.

[10] Tobias J, Vutukuru SR (2012). Simple and rapid multiplex PCR for identification of the main human diarrheagenic *Escherichia coli*. Microbiol Res.

[11] Vidal R, Vidal M, Lagos R, Levine M, Prado V (2004). Multiplex PCR for diagnosis of enteric infections associated with diarrheagenic *Escherichia coli*. J Clin Microbiol.

[12] Rodas C, Iniguez V, Qadri F, Wiklund G, Svennerholm A-M (2009). Development of multiplex PCR assays for detection of enterotoxigenic *Escherichia coli* colonization factors and toxins. J Clin Microbiol.

[13] Aranda KRS, Fabbricotti SH, Fagundes-Neto U, Scaletsky ICA (2007). Single multiplex assay to identify simultaneously enteropathogenic, enteroaggregative, enterotoxigenic, enteroinvasive and Shiga toxin-producing *Escherichia coli* strains in Brazilian children. FEMS Microbiol Lett.

[14] Toma C, Lu Y, Higa N, Nakasone N, Chinen I (2003). Multiplex PCR assay for identification of human diarrheagenic *Escherichia coli*. J Clin Microbiol.

[15] Vidal M, Kruger E, Durán C, Lagos R, Levine M (2005). Single multiplex PCR assay to identify simultaneously the six categories of diarrheagenic *Escherichia coli* associated with enteric infections. J Clin Microbiol.

[16] CLSI (2013). Performance Standards for Antimicrobial Disk and Dilution Susceptibility Tests for Bacteria Isolated from Animals; Approved Standard. VET01-A4.

[17] Regon M, Pathak DC, Tamuli SM, Baruah GK (2014). Serotyping of *Escherichia coli* isolated from piglet diarrhea. Vet World.

[18] Miliwebsky E, Schelotto F, Varela G, Luz D, Chinen I, Torres AG (2016). Escherichia Coli in the Americas.

[19] Gomes TAT, Elias WP, Scaletsky ICA, Guth BEC, Rodrigues JF (2016). Diarrheagenic *Escherichia coli*. Braz J Microbiol.

[20] Kotloff KL, Blackwelder WC, Nasrin D, Nataro JP, Farag TH (2012). The Global Enteric Multicenter Study (GEMS) of diarrheal disease in infants and young children in developing countries: epidemiologic and clinical methods of the case/control study. Clin Infect Dis.

[21] Mladenova Z, Steyer A, Steyer AF, Ganesh B, Petrov P (2015). Aetiology of acute paediatric gastroenteritis in Bulgaria during summer months: prevalence of viral infections. J Med Microbiol.

[22] Hossain A (2012). Presence and pattern of virulence genes in non-lactose fermenting *Escherichia coli* strains isolated from stools of children <5 years in rural and urban Bangladesh. Int J Infect Dis.

[23] Kahali S, Sarkar B, Rajendran K, Khanam J, Yamasaki S (2004). Virulence characteristics and molecular epidemiology of enteroaggregative *Escherichia coli* isolates from hospitalized diarrheal patients in Kolkata, India. J Clin Microbiol.

[24] Pabst WL, Altwegg M, Kind C, Mirjanic S, Hardegger D (2003). Prevalence of enteroaggregative *Escherichia coli* among children with and without diarrhea in Switzerland. J Clin Microbiol.

[25] Huang DB, Nataro JP, DuPont HL, Kamat PP, Mhatre AD (2006). Enteroaggregative *Escherichia coli* is a cause of acute diarrheal illness: a meta-analysis. Clin Infect Dis.

[26] Hebbelstrup Jensen B, Adler Sørensen C, Hebbelstrup Rye Rasmussen S, Rejkjær Holm D, Friis-Møller A (2018). Characterization of diarrheagenic Enteroaggregative *Escherichia coli* in Danish adults-antibiotic treatment does not reduce duration of diarrhea. Front Cell Infect Microbiol.

[27] Spano LC, da Cunha KF, Monfardini MV, de Cássia Bergamaschi Fonseca R, Scaletsky ICA (2017). High prevalence of diarrheagenic *Escherichia coli* carrying toxin-encoding genes isolated from children and adults in southeastern Brazil. BMC Infect Dis.

[28] Modgil V, Mahindroo J, Narayan C, Kalia M, Yousuf M (2020). Comparative analysis of virulence determinants, phylogroups, and antibiotic susceptibility patterns of typical versus atypical Enteroaggregative *E. coli* in India. PLoS Negl Trop Dis.

[29] Saha DR, Guin S, Krishnan R, Nag D, Koley H (2013). Inflammatory diarrhea due to enteroaggregative *Escherichia coli*: evidence from clinical and mice model studies. Gut Pathog.

[30] Cennimo D, Abbas A, Huang DB, Chiang T (2009). The prevalence and virulence characteristics of enteroaggregative *Escherichia coli* at an urgent-care clinic in the USA: a case-control study. J Med Microbiol.

[31] Huang DB, DuPont HL, Jiang ZD, Carlin L, Okhuysen PC (2004). Interleukin-8 response in an intestinal HCT-8 cell line infected with enteroaggregative and enterotoxigenic *Escherichia coli*. Clin Diagn Lab Immunol.

[32] Jindal N, Arora S (1991). Role of fecal leucocytes in the diagnostic evaluation of acute diarrhea indian. J Med Sci.

[33] McNeely WS, Dupont HL, Mathewson JJ, Oberhelman RA, Ericsson CD (1996). Occult blood versus fecal leukocytes in the diagnosis of bacterial diarrhea: a study of U.S. travelers to Mexico and Mexican children. Am J Trop Med Hyg.

[34] Steiner TS, Lima AAM, Nataro JP, Guerrant RL (1998). Enteroaggregative *Escherichia coli* produce intestinal inflammation and growth impairment and cause interleukin-8 release from intestinal epithelial cells. J Infect Dis.

[35] Greenberg DE, Jiang Z-D, Steffen R, Verenker MP, DuPont HL (2002). Markers of inflammation in bacterial diarrhea among travelers, with a focus on enteroaggregative *Escherichia coli* pathogenicity. J Infect Dis.

[36] Bhan MK, Bhandari N, Sazawal S, Clemens J, Raj P (1989). Descriptive epidemiology of persistent diarrhoea among young children in rural northern India. Bull World Health Organ.

[37] Fang GD, Lima AAM, Martins CV, Nataro JP, Guerrant RL (1995). Etiology and epidemiology of persistent diarrhea in northeastern Brazil: a hospital-based, prospective, case-control study. J Pediatr Gastroenterol Nutr.

[38] Rüttler ME, Renna NF, Balbi L, García B, Guidone L (2002). Characterization of enteroaggregative *Escherichia coli* strains isolated from children with acute diarrhea, in Mendoza, Argentina. Rev Argent Microbiol.

[39] Nataro JP, Kaper JB (1998). Diarrheagenic *Escherichia coli*. Clin Microbiol Rev.

[40] Sethabutr O, Venkatesan M, Murphy GS, Eampokalap B, Hoge CW (1993). Detection of *Shigellae* and enteroinvasive *Escherichia coli* by amplification of the invasion plasmid antigen H DNA sequence in patients with dysentery. J Infect Dis.

[41] Singh P, Metgud SC, Roy S, Purwar S (2019). Evolution of diarrheagenic *Escherichia coli* pathotypes in India. J Lab Physicians.

[42] Thakur N, Jain S, Changotra H, Shrivastava R, Kumar Y (2018). Molecular characterization of diarrheagenic *Escherichia coli* pathotypes: association of virulent genes, serogroups, and antibiotic resistance among moderate-to-severe diarrhea patients. J Clin Lab Anal.

[43] Chen LH, Hochberg NS, Magill AJ The Pretravel Consultation; Centre for Disease Control and Prevention. https://wwwnc.cdc.gov/travel/yellowbook/2018/the-pre-travel-consultation/the-pre-travel-consultation.

[44] O’Ryan M, Prado V, Pickering LK (2005). A millennium update on pediatric diarrheal illness in the developing world. Semin Pediatr Infect Dis.

[45] Dupont A, Sommer F, Zhang K, Repnik U, Basic M (2016). Age-dependent susceptibility to enteropathogenic *Escherichia coli* (EPEC) infection in mice. PLoS Pathog.

[46] Natarajan M, Kumar D, Mandal J, Biswal N, Stephen S (2018). A study of virulence and antimicrobial resistance pattern in diarrhoeagenic *Escherichia coli* isolated from diarrhoeal stool specimens from children and adults in a tertiary hospital, Puducherry, India. J Health Popul Nutr.

[47] Chellapandi K, Dutta TK, Sharma I, De Mandal S, Kumar NS (2017). Prevalence of multi drug resistant enteropathogenic and enteroinvasive *Escherichia coli* isolated from children with and without diarrhea in Northeast Indian population. Ann Clin Microbiol Antimicrob.

